# National Thoracic Surgery Standards Implementation: Barriers, Enablers, and Opportunities

**DOI:** 10.3390/curroncol28010043

**Published:** 2021-01-13

**Authors:** Angel Arnaout, Anubha Prashad, Nadine Dunk, Jess Rogers, Annemarie Edwards, Mary Argent-Katwala, Christian Finley

**Affiliations:** 1 Canadian Partnership Against Cancer, 145 King Street West, Toronto, ON M5H 1J8, Canada; Anubha.Prashad@partnershipagainstcancer.ca (A.P.); Nadine.Dunk@partnershipagainstcancer.ca (N.D.); jess.rogers@partnershipagainstcancer.ca (J.R.); Annemarie.Edwards@partnershipagainstcancer.ca (A.E.); mary@notcontrary.com (M.A.-K.); Christian.Finley@partnershipagainstcancer.ca (C.F.); 2 Department of Surgery, University of Ottawa, Ottawa, ON K1H 8L6, Canada; 3 Department of Surgery, McMaster University, Hamilton, ON L8S 4K1, Canada

**Keywords:** Thoracic, surgery, standards, cancer, implementation, barriers, enablers

## Abstract

Background: Diagnosis and surgical treatment decision making for thoracic cancers is complex. Moreover, there is demonstrated variability in how each province in Canada delivers cancer care, resulting in disparities in patient outcomes. Recently, the Canadian Partnership Against Cancer (CPAC) published pan-Canadian evidence-based standards for the care of thoracic surgery cancer patients. This study was undertaken to assess the degree to which these standards were currently met in practice and to further understand the determinants to their implementation nationally. Methods: This study was undertaken in two parts: (1) a national survey of thoracic surgeons to assess the perceived extent of implementation of these standards in their institution and province; and (2) formation of a focus group with a representative sample of thoracic surgeons across Canada in a qualitative study to understand the determinants of successful standards implementation. Results: 37 surgeons (33% response rate) participated in the survey; 78% were from academic hospitals. The top categories of standards that were under-implemented included (a) quality assurance processes, data collection and clinician audit and feedback, and (b) ongoing regional planning and workload assessments for thoracic surgeons, and (c) pathology turnaround time target of two weeks and the use of a standardized synoptic pathology report format. Enablers, barriers, and opportunities for standards implementation contextualized the discussion within the focus group. Conclusion: Study results demonstrated variation in the implementation of surgery standards across Canada and identified the determinants to the delivery of high quality surgical care. Future work will need to include the promotion and development of quality improvement strategies and effective resource allocation that is aligned with the implementation of thoracic cancer surgery standards in order to improve patient outcomes.

## 1. Background

As in the rest of the world, cancers of the thorax, mostly comprised of lung and esophageal cancer, are the leading cause of cancer related mortality [1]. On a population of almost 37 million inhabitants in Canada, over 28,000 persons were diagnosed with lung cancer and an estimated 2300 were diagnosed with esophageal cancer in Canada in 2017 [2]. Despite the significant decrease in cancer mortality over the past 20 years, thoracic cancers remain the leading cause of cancer-related death in Canada, killing more people than prostate, colon, and breast cancers combined [3].

Findings from a special report from the federally sponsored, Canadian Partnership Against Cancer (CPAC) in 2015 demonstrated tremendous variability in how each province in Canada delivers cancer care services, resulting in disparities in patient outcomes [4]. Overall age adjusted in-hospital mortality ranged from 1.5% to 3.8% for lung cancer and 2.7% to 11% for esophageal cancer across Canada. This stark variability in thoracic cancer outcomes culminated in the development of pan-Canadian standards for thoracic surgery by CPAC, a federally funded organization [3]. These standards highlight the required training qualifications of the treating surgeons, necessary resources to consistently deliver proper care in the treatment facilities, and the minimum quality control processes needed for the high quality care at an institutional, regional, and provincial level.

CPAC investigators undertook a two-part study to mobilize implementation of thoracic surgery standards. The first part of the study included a national survey of thoracic surgeons to assess the extent of implementation of the evidence-based standards in their institution and province. The second part of the study consisted of the formation of a focus group with a representative sample of thoracic surgeons across Canada to further understand the barriers and facilitators to implement thoracic surgery standards.

## 2. Methods

### 2.1. Standards

#### 2.1.1. Study Design

We developed an electronic survey that assessed the degree of implementation of each item in the thoracic surgery standards document [3], as perceived by the surgeon. Questions asked the surgeons to rate the extent of standards implementation for (a) their institution and (b) their province based on a Likert scale consisting of five categories. Surveys (administered through online survey platform QuestionPro Inc., Austin, TX, USA) were sent out via email membership mail-out from the Canadian Association of Thoracic Surgeons (CATS, 113 members in 2019). Survey reminders were sent out 4 times over a period of 2 months. Surgeons had to be members of CATS, actively treating patients with thoracic cancer, and have agreed to participate with informed consent. Survey participation email included instructions for respondents (e.g., respondents were unable to change their response once submitted; and each visitor was only able to complete the survey once) (Please see Supplemental Materials for the Checklist for Reporting Results of Internet E-Surveys (CHERRIES) and content of surveys).

#### 2.1.2. Survey Data Analysis

For each standard, survey respondents were asked to rate the extent of compliance within their center as well as their province (using a 5-point Likert-type scale). They were also provided with the opportunity to provide text comments throughout the survey. All survey responses were de-identified prior to analysis.

Frequencies of response categories were calculated and displayed as stacked bar charts to show the variation in the extent to which each standard was implemented in the surgeon’s institution and province. The target for implementation was established as 70% (i.e., of respondents answering “to a great extent”); this target was selected by the expert panel that contributed to the development of the thoracic cancer surgical standards [3]. Based on the results of the survey, we identified the top three categories of standards which demonstrated the lowest degree of implementation either at the institution and/or provincial level. These categories of standards were then discussed at the focus group (Supplemental Materials).

### 2.2. Focus Group to Assess Enablers and Barriers to Implementation of Thoracic Surgery Standards

#### 2.2.1. Study Design

The focus group was conducted during the annual CATS meeting on 13–15 September 2018, in St Johns, Newfoundland. A purposeful and stratified (by region) sampling strategy was used to select up to 15 surgeons from those already registered to attend the meeting and specialised in thoracic surgery. We attempted to recruit surgeons representative of each Canadian province and territories if possible. All eligible surgeons were sent a recruitment email inviting them to the focus group. Those who displayed interest in participating in the study were sent information on the study including a study consent form. Informed consent was obtained from all participants as per ethical guidelines prior to any collection of data. The Supplemental Materials includes more details around the methods used for the focus group including the COREQ checklistas well as a discussion guide to assess the enablers and barriers faced by the surgeons as well as opportunities in regard to the implementation of each of the standards. 

#### 2.2.2. Data Collection and Analysis

Digital recordings from the focus groups were transcribed verbatim, imported into the qualitative data analysis software NVivo (version 1033) and verified by the study team prior to analysis. This qualitative descriptive study used direct content analysis of data from the focus group. We aimed to identify the determinants that impacted the degree of standards implementation. These determinants were categorized as either enablers, barriers, or opportunities, and then further categorized into individual (surgeon), organizational, or system (regional/provincial) level determinants. Individual determinants illustrated physician intentions, knowledge, comfort level and routine practices. Organizational determinants captured determinants that were Influenced from hospital or cancer center structure, policies and priorities, resource forces, and management and organizational culture. Finally, system determinants reflected healthcare system structures and processes. We used a deductive approach to qualitative analysis—Analyzing the data based on a predetermined coding structure. Initial categories for coding the focus group were developed by the authors, and sub-categories were noted when reading through the focus group transcripts.

Data analysis occurred in 2 steps: (1) coding was performed by 4 team members (AA, JR, ND, and AP) in independent manner, for each category of standards discussed; (2) the 4 members then met frequently to compare their coding and discuss discrepancies until consensus was achieved. Theming was then performed by grouping determinants that were similar in concept for each category of standards. Opportunities were noted if potential interventions to support the implementation of surgical standards emerged during the process of coding and theming.

## 3. Results

### 3.1. National Survey

Thirty-seven respondents participated in the survey (33% of CATS membership) from Eastern Canada (*n* = 7, 19%), Western Canada (*n* = 13, 35%), Quebec (*n* = 4, 11%), and Ontario (*n* = 13, 35%). Of these, 46% of surgeons were in their earlier years of practice (0–10 years), 27% in mid-years (11 to 20 years), and 27% in their later years of practice (20+ years). In addition, 22% of surgeons were from community hospitals while 78% were from academic hospitals.

The results of the survey demonstrated areas in which there were significant gaps in compliance with the standards (see Supplementary Materials). The top three categories of standards that were most under-implemented included (a) quality assurance processes, data collection and clinician audit and feedback, (b) regional planning and workload assessments for thoracic surgeons, and (c) pathology turnaround time target of two weeks and the use of a standardized synoptic pathology report format. These formed the basis for the discussion in the focus groups. All surveys were analyzed regardless of their completion ratio.

### 3.2. Focus Group

Discussion of the previously mentioned two categories of standards resulted in the identification of 81 determinants (enablers and barriers). For each standard, the determinants fell into three main categories: individual physician (*n* = 12, 15%); organizational (*n* = 33, 41%), and system (*n* = 36, 44%).

#### 3.2.1. Surgical Standards Category 1: Data Driven Quality Improvement Processes Including Collection of Patient Outcomes Data and Clinician Audit and Feedback

##### Sample Quotes

“I think the biggest problem with collection and measuring data is having a person to do it and that’s what we do not get in the hospitals. If you want someone to collect data for the national database, you need a full-time person who’s diligent. And at the hospital, they don’t support you at all for that […] there’s always no money, no money, no money.”

“I suspect most hospitals in some capacity collect this type of data but it’s far from what we need as a robust data as an audit and feedback tool, that’s for sure. It’s more like, ’How was the food? How was the parking?’ […] which is important stuff for people. […] but that certainly doesn’t in any meaningful way give us useful feedback.”

##### Enablers

Individual surgeon motivation for data collection and data-driven improvement processes were considered key enablers by participants of the focus group. The importance of hard data points (e.g., mortality and length of stay), including use of patient experience, quality of life and satisfaction data, was emphasized in building the case for why quality assurance processes are important. In a few provinces, such as Alberta, surgeons are incentivized to participate in data entry and quality improvement processes with the use of Continuing Medical Education (CME) credits, which were noted by the participants as being helpful. An organization-level enabler that was noted was the alignment of organizational incentives with data collection initiatives (e.g., collection of surgical wait time data was easily facilitated and incentivized if it was an organizational priority). To this end, some participants noted that their hospitals have dedicated decision support teams for specific types of data extraction and analysis.

##### Barriers

While participants recognized that data collection is important, they noted that there is no consistent data holding and/or technical definitions used nationally. In addition, there is a lack of infrastructure, software, and human resources in supporting data collection and reporting. At the individual level, physician workload and burnout were reported as significant barriers to sustained efforts at data collection and quality improvement initiatives. Another barrier noted by the participants was that often care delivery decisions are based on what the organization is measured on and accountable for (e.g., wait times), and some of the target benchmarks for the various metrics are not evidence based or actually thought to be what is best for the patient. Overall, while all participants agreed that while the CPAC standard of robust data collection and its use for quality improvement processes was likely to improve patient outcome, the cost of implementing such a standard at many hospitals would be substantial which makes this CPAC standard a “nice to have” versus a “must have”.

##### Opportunities

Participants articulated opportunities to help facilitate the implementation of this CPAC standard. These included engaging in a cost-benefit analysis to support recruitment of additional staff to cover non-clinical responsibilities such as data collection. For example, analysis around linking higher hospital case volume with reduced wait times can warrant recruitment of additional manpower. In addition, with respect to data collection, there is an opportunity to explore the use of a core minimum data set with supporting technical definitions that can be consistently used across jurisdictions. Quantitative data combined with patient voice, endorsement by national professional societies and partnership with Accreditation Canada can further strengthen the case of why quality assurance processes are important for patient care.

#### 3.2.2. Surgical Standards Category 2: Regional Forecasting of Manpower Needs and Workload Assessment of Thoracic Surgeons

##### Sample Quotes

“So you look at ministry in the face and say, ‘Okay, there’s only two of us right now. So you expect me every other night that I have to be on call for the rest of my life? Do you think that’s good for me? Do you think that’s good for me and the patient?’”

“For a small province—to give you an example, I am good friends with physician lead for the Saskatchewan Health Authority. So because I know her, we’ve got funding for another physician.”

“It’s so difficult to actually prove that bringing in another person will be beneficial to the hospital. It comes down to doing cost analysis. What is the effect in terms of the OR time and endoscopy, where are we going to put their office space? That’s what it comes down to if you’re trying to increase the numbers […] plus they’ll tell you […] there’s no extra OR time—you’re dividing it up [amongst yourselves].”

##### Enablers

Several jurisdictional examples were shared by participants where recruitment of additional staff helped addressed surgeon workload concerns. Some organizations noted hiring of nurse managers to address administrative needs and patient navigators to streamline the patients’ post-operative transition to home and community settings as key enablers. From a system level, workforce planning is dependent on local circumstances and patient advocacy groups can help provide a quality case to recruit additional surgeons to meet the unmet needs of the region.

##### Barriers

Participants reported local politics as barriers to appropriate provision of the necessary manpower to complete the thoracic surgery volumes and on-call clinical duties. Physicians often cannot take vacation due to the lack of coverage. In addition, physician funding plans do not adequately account for non-clinical responsibilities, such as research, teaching, and administration. These responsibilities are often not integrated into workload when recruiting new surgeons. From a system level, participants thought that global budgets do not effectively compensate for the quality of services and care provided by thoracic surgeons.

##### Opportunities

An opportunity discussed included a pan-Canadian organization, such as CPAC, to collaborate with ministerial health authorities and cancer agencies to explore alternate physician funding plans. This may help alleviate workload balance and staffing concerns.

#### 3.2.3. Surgical Standards Category 3: Pathology Turnaround Time Target of Two Weeks and the Use of a of a Standardized Synoptic Pathology Report Format

##### Sample Quotes

“Getting pathology quickly […] is about getting people to treatment quickly”.

“We had it [two week turnaround time for pathology], we had three thoracic pathologists. One decided to leave. They were interviewing for the spot and the hospital. No, the hospital decided that we can’t afford it. So they closed it so now our wait times is four to six weeks and the hospital, I mean […] it is what it is […] they don’t really seem to care.”

“It’s not just the pathologists; it’s the technicians…the people that prepare the pathology […] because with us, that was the problem. They did hire a couple of pathologists and now it’s not a pathologist issue anymore […] or that’s what they say […].”

##### Enablers

The presence of dedicated thoracic pathologists was noted as an enabler of reducing pathology turnaround times and the use of synoptic pathology reports within an organization. In addition, synoptic template for pathology reporting was identified as a key enabler in providing rapid and meaningful pathology reports that would not only help in treatment decision making but also would be easier to use for organizational quality improvement processes.

##### Barriers

Participants noted that a major barrier to meeting the two week turnaround time target for pathology was if their organizations did not try to proactively or retroactively match the number of thoracic surgeons to the number of available thoracic pathologists/ pathology technologists when it came to recruitment of surgeons. In addition, national pathology standards do exist for pathology turnaround times, but participants felt that there was no accountability within their organizations to meet those standards.

##### Opportunities

Participants highlighted the opportunity to engage in multi-disciplinary discussions with pathology, radiology, and surgery and hospital leadership to ensure adequate pathology manpower exists to achieve this standard and to manage patient/hospital/physician expectations for pathology turnaround times.

## 4. Discussion

Deliberate approaches are needed to improve the organization of complex surgeries in a way that optimizes patient outcomes and reduces the burden on health care resources. Oncologic quality is an assessment of the value of the various aspects of medical care provided to a patient from their first contact with a physician through completion of their care. Organizations such as the Canadian Association of Thoracic Surgeons (CATS), the American College of Chest Physicians (ACCP), the British Thoracic Society (BTS), the European Society of Thoracic Surgeons (ESTS), the American College of Surgeons (ACS), the National Comprehensive Cancer Network (NCCN), and the Society of Thoracic Surgeons (STS) have all proposed, endorsed, and intermittently updated treatment guidelines aimed at improving the quality, effectiveness, and efficiency of cancer care worldwide. These guidelines often represent a combination of best available evidence and expert opinion [5,6,7,8,9,10,11]. Despite these efforts, a review of the worldwide literature reveals a variable level of alignment between what the organizations recommend, what the institution supports, and what the clinician practices.

In Canada, cancer surgical quality is integrated in the role of provincial cancer agencies and as such a national unified approach to cancer surgical care in general, is lacking. When it comes to thoracic surgery, clear variations exist across the country in patient outcomes [4,12,13,14,15]. As a result of this, CPAC, a federally funded organization, took on the task of developing evidence-based national standards in thoracic cancer surgery which have been recently endorsed by the CATS [3]. This study was therefore undertaken to understand the current practice and the determinants to the appropriate use of surgical standards by all stakeholders. This is a crucial first step in designing effective strategies aimed at individual physicians, organizations, and the health care system.

Health care quality can improve through changes in policy or behavior at all levels (federal government, provincial and territorial governments, regional health authorities, health care delivery organizations, individual clinicians, and patients) [16]. Ontario’s advisor on quality in healthcare, Health Quality Ontario, noted that ways to improve the quality of care include expanding public funding for proven treatments, investing in primary care, and embedding tools of improvement into practice [16]. They also emphasized that improvements in care is more likely with the involvement of both front-line clinicians and patients. The issue of embedding quality improvement tools into practice is commonly in the literature on quality. A recent study assessing the quality of cardiac care in Canada gave three recommendations when it comes to building a sustainable high quality healthcare system [17] (1) that funding be secured for the provincial collection of quality indicators and the creation of annual national quality reports; (2) that the culture of using quality indicator data for continuous quality improvement and national/regional sharing of best practices be enhanced and (3) that ongoing evaluation and revision of clinical practice guidelines incorporating key quality indicators be implemented. Interestingly, an analysis of the survey results revealed the top three categories of standards that were under-implemented: (a) quality assurance processes, data collection and clinician audit and feedback (b) regional planning and workload assessments for thoracic surgeons and (c) pathology turnaround time target of two weeks and the use of a standardized synoptic pathology report format.

### 4.1. Quality Assurance Processes, Data Collection, Clinician Audit and Feedback

The most cited barriers for data collection and reporting were incentives and resources. It was clear that data collection was more likely to occur if it aligned with organizational priorities (such as wait time to diagnosis or treatment) or if the individual physicians or organizations were accountable to the cancer jurisdiction regarding compliance with certain quality metrics or indicators.

Audit and feedback of performance data has been recognized as a valuable tool to assess and improve the quality of care [18]. The provincial surgical synoptic reporting initiative in Alberta, Canada, for example, has made it easy for surgeons to enter data and receive feedback regarding their practices in comparison to their peers. In addition, Ontario has initiated a Surgical Quality Improvement Network, where local communities of practice groups have enabled surgeons to receive individual report cards regarding their performance on quality indicators that were deemed relevant by the surgeons to measure. In the Netherlands, the Dutch Lung Surgery Audit has created a platform for clinicians to develop new meaningful quality indicator and benchmarks, thereby stimulating improvement initiatives on both local and national level. Benchmarked information on hospital performance is returned to clinicians to enable them to improve patient outcomes [19,20]. This information is presented in the form of quality of care indicators that are derived from the data collected in the audit and can be used to make (variation in) quality of health care more transparent. Implementation of evidence-based guidelines and quality standards is evaluated with the audit, on a local as well as a national level. The incentive for clinicians to participate in the audit is the information they receive on the quality of their performance in clinical practice with indicator results benchmarked to the national average (intrinsic motivation). In addition, The Netherlands Healthcare Inspectorate demands participation in the audit, insurance companies use the audit information for reimbursement and the National Healthcare Institute demands indicator scores from the audit for public transparency, which makes participation more or less mandatory for hospitals (external stimulus). One of the solutions to reduce administrative burden is (partly) automated data extraction from existing data sources (e.g., electronic patient records (EPDs), structured reports of diagnostics, treatment, or pathology). Being part of a larger platform, such as NSQUIP [21], can be an advantage in this, when close cooperation is sought between the registry platform, the data processor, and hospital-IT-provider [20].

### 4.2. Regional Workforce Planning and Workload Assessments for Thoracic Surgeons

Accurately forecasting workforce requirements has become increasingly important in recent years, as government agencies work to ensure appropriate resource allocation and as specialty societies attempt to predict workforce needs [22,23,24]. Determining workforce needs for surgeons is a complex process that is influenced by several factors, including the age and sex of active surgeons, estimates of migration by active surgeons in and out of jurisdictions, rates of surgical procedures, current and future scope of practice, and overall population growth and aging [21]. A survey of Canadian thoracic surgeons in 2013 demonstrated that one-third of respondents felt there were too few surgeons. Most respondents (58.5%) felt that a combination of measures should be used to estimate thoracic surgery workforce requirements, whereas 24.5% indicated that using the ratio of one thoracic surgeon per X population was the most useful measure to predict workforce needs. For those that indicated a preference for a ratio of full-time thoracic surgeons to number of people in the population, most respondents (51.1%) felt that one thoracic surgeon per 250,000 people was the most appropriate ratio in Canada. No respondents felt that the ratio should be greater than 1:500,000. Furthermore, an analysis of members of CATS estimated that on average, each full-time equivalent thoracic surgeon provides service to 150,000–500,000 people [25]. Clearly the issue of thoracic surgeon manpower management needs to balance safety for thoracic surgery patients versus cost to the organization and healthcare system.

In Canada, thoracic cancer surgery is usually practiced in large urban academic centers, in association with a regional cancer center and trauma center. This model of regionalized care is important for reducing duplication of resources and establishing multidisciplinary groups with expertise in thoracic cancer and trauma care, as evidence suggests that such high-volume assessment and operative teams improve patient outcomes and quality of care [26]. Furthermore, ideally these centers would support a core group of thoracic surgeons in maintaining a good work–life balance by allowing an appropriate on-call schedule and allowing time off for continuing medical education. Despite many efforts to implement this, our recent focus group analysis demonstrated that surgeon burn out is still a significant issue, as some surgeons stated that a persistent lack of manpower is often an obstacle to surgeons taking time off. Beyond the negative effect that burnout has on individual physicians, their families, and their colleagues, burnout also has severe effects on the quality of care provided to patients, with increased likelihood of committing a major medical error [27]. In 2009, a survey from the American College of Surgeons to its members (*n* = 24,922) revealed that overall 40% of surgeons experienced burnout, and amongst the most affected are those that are front line surgical subspecialties, such as trauma, thoracic, vascular, and general surgery [27]. In addition to subspecialty, this study also demonstrated that in addition to subspecialty, other factors that were significantly associated with burnout on multivariate analysis included having young children (≤21 years), volume incentivized (fee-for-service) payment model, non-physician spouses in health care, number of call nights per week, hours worked per week, and years in practice. Conversely, whereas physician age, having children, and having greater than 50% of time dedicated to non-patient care activities were all protective. Interestingly, participants in the focus group noted that a surgeon’s potential administrative activity should be factored into the calculation of thoracic surgeon human resource workforce requirements. Sole emphasis on the projected clinical workload as a method to estimate the number of thoracic surgeons needed would likely result in administrative duties added on top of a surgeon’s heavy clinical load, leading to a higher likelihood of burnout. Finally, although volume incentivized payment models are associated with burnout in the ACS study, there exists a paucity of studies in the literature that examine whether alternate payment models (salaried and bundled payments) actually improve work–life balance and reduce burnout in physicians in general. What seems to be clear in the literature is that financial stress is connected to burnout; physicians do respond to financial incentives; and volume-incentivized payment models encourage physicians to overwork, especially those physicians with pre-existing significant financial debt. An additional barrier to resolving this issue is that among physicians, honest discussions of the impacts of physician compensation are often taboo because of the inherent tension between the altruistic associations of patient care and the financial realities of medical practice.

### 4.3. Two Week Pathology Turnaround Time and Synoptic Pathology Reporting

Turnaround time, defined as time from specimen collection to production of a report, has always been an important quality indicator not only in pathology but also quality and safety of patient care [28]. Hence, verifying pathology reports in an appropriate time frame helps physicians with diagnosing patients in a timely fashion, which will not only lead to an effective treatment plan but also allows for faster initiation of treatment [29]. Research evidence shows that a prolonged turnaround time can raise increase morbidity and mortality rates, such as with oral cancers [30]. Although there is variation in the individual institutional targets for turnaround times, known international authorities such as College of American Pathologists (CAP), which is also endorsed the American Society of Clinical Oncology (ASCO), recommend that most routine cases (≥90%) should have a turnaround time of two days or less for the surgical pathology report [29,31]. Most surgeon participants in this study felt that their experience with post-surgical turnaround times was even longer than the two weeks as recommended by CPAC. In addition to ensuring adequate human resources (pathologists and pathology technicians), helping resolve this difference requires an in-depth mapping out of the processes that make up the turnaround time that may be expedited or performed in parallel, or removed if redundant. These processes span through the three phases of specimen reporting, including the pre-analytic (specimen delivery, identification, and fixation), analytic (immunohistochemistry, histology, and molecular testing analyses by the pathologist) and post analytic (transcription and report verification) [31,32].

### 4.4. Overall Study Strengths and Limitations

This study has some notable strengths. First, this is the first Canadian national evaluation of the determinants of standards implementation in thoracic cancer surgery. The surgeon participants had good representation across sex, age, and years in practice. Secondly, the qualitative approach undertaken allowed for a detailed analysis of the determinants necessary to inform strategies to implement high quality thoracic cancer surgery nationally.

The current study also has some limitations. For the survey responses, there could be selection bias from the surgeons who answered the survey and/or came to the focus group; in that, they may be representative of the opinions of their colleagues. Our opt-in sampling strategy may mean that we captured individuals with the strongest views (either positive or negative) on the topic and who are most passionate about sharing them, meaning that we may have missed some valuable feedback from those who are more impartial. In addition, we were only able to measure perceived implementation of standards from the participants which may be different from reality. This is especially true when discussing standards implementation at the provincial level, as some surgeons may not have reliable knowledge at this level. Furthermore, while the study used surgeons from a variety of geographic locations throughout Canada, 78% were from academic hospitals. This means that the views shared by the thoracic surgeon participants may not be generalizable to the community surgeon setting. Finally, while thoracic cancer surgical care is multidisciplinary, not all members of the multidisciplinary thoracic team were, such as pathologists, radiologists, and oncologists. Incorporating the views of these groups may provide a potentially fuller picture of the determinants to standards implementation in thoracic cancer surgical care.

## 5. Conclusions

Overall, the results of this study allowed the investigators of CPAC to recognize the variation in the implementation of surgical standards across Canada and identify potential barriers to delivery of high quality thoracic cancer care. Future work will need to include the promotion and development of quality improvement strategies and effective resource allocation that is aligned with implementation of the pan-Canadian thoracic surgery standards in order to improve patient outcomes.

## Supplementary Materials

The following are available online at https://www.mdpi.com/1718-7729/28/1/43/s1, Table S1. Checklist for Reporting Results of Internet E-Surveys (CHERRIES). Questionnaire S1. Pan-Canadian Standards for Thoracic Cancer Surgery-Compliance Survey. Table S2. Table of Survey Results for Standards with lowest implementation for each institution and province. Questionnaire S2. Consolidated criteria for reporting qualitative studies (COREQ): 32-item checklist. Questionnaire S3. Focus Group Questions and Discussion Guide.
