# Supplementary material for: National Thoracic Surgery Standards Implementation: Barriers, Enablers, and Opportunities
Source: Curr Oncol. 2021 Jan 13;28(1):405–16. doi: 10.3390/curroncol28010043 (PMC7903269; doi:10.3390/curroncol28010043)
Supplement: Supplementary file 1 [file curroncol-28-00043-s001.pdf]

CURRENT ONCOLOGY
A Canadian Cancer Research Journal

Supplemental Materials for

National Thoracic Surgery Standards Implementation: Barriers, Enablers & Opportunities

Listing of Supplemental Materials:

Table S1: Checklist for Reporting Results of Internet E-Surveys (CHERRIES)

Questionnaire S1: Pan-Canadian Standards for Thoracic Cancer Surgery - Compliance Survey

Table S2: Table of Survey Results for Standards with lowest implementation for each institution and province

Questionnaire S2: Consolidated criteria for reporting qualitative studies (COREQ): 32-item checklist

Questionnaire S3: Focus Group Questions and Discussion Guide

Table S1: Checklist for Reporting Results of Internet E-Surveys (CHERRIES)[5]

Item Category	*Checklist Item*	*Explanation*	*Location*
**Design**	Describe survey design	Surveys were sent out via email membership mail-out from The Canadian Association of Thoracis Surgeons (CATS). Surgeons had to be actively treating patients with thoracic cancer and had to have agreed to participate with informed consent. A convenience sample was used.	Methods section
**IRB Approval and Informed Consent Process**	IRB approval	Institutional ethics approval was obtained	Methods section
	Informed consent	It was a voluntary survey. The participants were told the length of time of the survey (10-15 minutes). The investigators were named. The purpose of the study was explained in the invitation email. The email also explained that all responses were confidential and anonymous and that reporting would be on an aggregate level only. Consent was indicated when respondents clicking the 'Go to Survey' button from this page.	Methods section
	Data protection	Proprietary survey software was used to ensure data protection. No personal information was linked to survey results in any way. The fully de-identified dataset is kept on password protected computers.	Methods section
	Development and testing	The survey was developed from the published pan-Canadian evidence-based surgical standards for the care of the thoracic cancer patient (1) developed by CPAC. The usability and technical functionality was cognitively tested by 2 surgeons.	Methods section
	Open survey versus closed survey	The survey was a closed survey. Access was provided to only those that received the invitation email.	Methods section

Contact mode	Participants were contacted by email only.	Methods section
Advertising the survey	The survey was not advertised.	Methods section
Web/E-mail	The surveys were administered through an online software, QuestionPro. The survey was not posted on a Web site. Access to the survey was directly from the email invitation once the patient performed an electronic informed consent.	Methods section
Context	The survey was not posted on a Web site.	Methods section
Mandatory/voluntary	It was a voluntary survey.	Methods section
Incentives	There were no incentives offered.	
Time/Date	Survey reminders were sent out 4 times over a period of 2 months	Methods section.
Randomization of items or questionnaires	Randomization of items/questionnaires was not performed.	Methods section
Adaptive questioning	Adaptive questioning was not used as the questions required the participants to rate the extent of standards implementation based on a Likert scale, with the option of adding comments to each answer.	Methods section
Number of Items	There were 1 to 4 questions per page depending on the length of the question.	Methods section<br>Appendix 3
Number of screens (pages)	The questions were distributed over 10 pages.	Appendix 3
Completeness check	All survey items were deemed to be mandatory, and respondents prompted to complete outstanding items before leaving the survey page on which the item was	Appendix 3

	contained.	
Review step	Respondents were unable to change their responses once submitted.	Methods section
Unique site visitor	Each visitor was only able to complete the survey once.	Methods section
View rate (Ratio of unique survey visitors/unique site visitors)	The survey was not posted on a Web site (email invitation only).	Method section
Participation rate (Ratio of unique visitors who agreed to participate/unique first survey page visitors)	This was named response rate in the paper. The denominator corresponded to the number of invited surgeons within the CATS membership.	Results section
Completion rate (Ratio of users who finished the survey/users who agreed to participate)	N/A	N/A
Cookies used	Cookies were not used because respondents were recruited from an existing sampling frame.	Methods section
IP check	IP address was not used to identify duplicate entries.	Methods section
Log file analysis	N/A	N/A
Registration	Entry to the survey for each participant was via a unique link from the e-invitation.	Methods section
Handling of incomplete questionnaires	All questionnaires were analyzed, whether they were complete or incomplete.	Results section
Questionnaires submitted with an atypical	No respondents were removed from the survey for	Results section

	timestamp	completing the items too quickly.	
	Statistical correction	No statistical correction was used.	Results section

Questionnaire S1: Pan-Canadian Standards for Thoracic Cancer Surgery - Compliance Survey

The Pan-Canadian Standards for Thoracic Surgery seeks to elevate the delivery of thoracic surgical care in Canada. The document provides high-level guidance and discussion on the foundational resources and requirements that need to be in place to improve cancer surgical care and outcomes. The document has been endorsed by the Canadian Association of Thoracic Surgeons (CATS) and was officially released at the end of March 2018. The purpose of this survey is to assess compliance of the standards at institutions across Canada, as well as at the provincial-level. We kindly ask that you review the standards and assess if each standard is currently being met at your centre and in your province. At the end of the survey, you will have an opportunity to provide additional information. This survey should take approximately 10-15 minutes to complete.

We would like to thank you for your time and participation in helping us assess pan-Canadian compliance. Upon your feedback, we will collate and anonymize all responses and use this data to assess gaps across the country and refine our implementation strategy.

Noted below are a few helpful tips to complete the survey:

- Each standard will be presented in turn in the survey.
- For each standard, you will be asked if your centre and province are currently in compliance.
- You can select 1 of 3 options: Yes Partial No
- You will have the opportunity to also provide text comments throughout the survey. Please feel free to save the completed portions of the survey and come back to it at your convenience. Should you experience any technical issues with this survey, please contact us at quality@partnershipagainstcancer.ca to troubleshoot or recover responses.

DEMOGRAPHICS

1. Name

2. Province/Territory of Work (please select only one):
    1. British Columbia
    2. Alberta
    3. Saskatchewan
    4. Manitoba

5. Ontario
6. Quebec
7. New Brunswick
8. Nova Scotia
9. Prince Edward Island
10. Newfoundland and Labrador
11. Yukon
12. Northwest Territories
13. Nunavut

3. Name of Hospital(s)/Cancer Center you are affiliated with?

4. Where do you work?
   1. Community Hospital
   2. Academic Hospital

5. What is your primary role?
   1. Clinician- Surgeon
   2. Clinician- other
   3. Administrator
   4. Researcher
   5. Other __________

6. If you are a clinician, what percent (%) of your practice is dedicated to Thoracic Surgery?

7. Number of years in practice since latest graduation (from residency or fellowship)?

8. Number of thoracic surgeons at your center?

9. Approximate number of thoracic surgeries performed per year in your center?

STANDARDS

1. **SURGEON CRITERIA**

1.1 TRAINING AND MAINTENANCE OF COMPETENCIES

Please comment on the extent to which the following standards are being met in your a) institution b) province:

Standards	Your Institution					Province					Comments
	To a great extent	Some what	Very little	Not at all	Don't know	To a great extent	Some what	Very little	Not at all	Don't know	

1.1.1 Currently, a practicing thoracic surgeon has contemporary knowledge of the diseases of the thorax and foregut as defined by the objectives of training in the Subspecialty of Thoracic Surgery in the Royal College of Physicians and Surgeons of Canada.			
1.1.2 Currently, thoracic surgeons are mandated to participate in the maintenance of competency in accordance with provincial and national standards.			
1.1.3 Currently, thoracic surgeons have formal, complete and certified training in thoracic surgery equivalent with Royal College of Physicians and Surgeons of Canada (RCPSC). For those not trained in Canada, a similar regimented and accredited training program has been completed.			

1.2 SURGERY & MANAGEMENT

Standards	Your Institution					Province					Comments
	To a great extent	Some what	Very little	Not at all	Don’t know	To a great extent	Some what	Very little	Not at all	Don’t know	
1.2.1 Currently, thoracic surgeons are intimately involved in the diagnostic assessment											

and management of benign and malignant lung, esophageal and other thoracic tumours, where only the thoracic surgeon			
Currently, the decision of operability and resectability of a cancer is made only by the thoracic surgeon			
1.2.2 Currently, resections for lung cancer and esophageal cancer are performed by thoracic surgeons in designated thoracic surgical centers.			

2. PRACTICE SETTINGS

2.1 ORGANIZATIONAL CRITERIA

Standards	Your Institution					Province					Comments
	Yes		No		Don't know	Yes		No		Don't know	
2.1.1 Recognizing regional needs, currently, a thoracic center has at least three thoracic surgeons at each designated center											
	To a great extent	Some what	Very little	Not at all	Don't know	To a great extent	To a great extent	Very little	Not at all	Don't know	
Currently, recruitment of additional **thoracic surgeons** is based on certain factors or thresholds such as increase in non-clinical responsibilities (education, research or leadership); workload; referrals; wait times.											
Currently, recruitment of additional **human resource supports** is based on certain factors or thresholds such as increase in non-clinical responsibilities (education, research or leadership); workload; referrals; wait times.											
2.1.2 Currently, thoracic centers set targets to monitor and evaluate wait times and timely access to care.											

2.1.3 Within the geographic limitations of a health authority, specialized services are concentrated and regionalized.											
2.1.4 Currently, thoracic centers participate in integrated and established networks of care to ensure appropriate care is provided closer to home (regionally/ provincially).											
2.1.5 Infrastructure is currently in place to support the participation of patients in clinical research.											

2.2 PHYSICAL RESOURCES AND COLLABORATING SERVICES

Standards	Your Institution					Province					Comments
	To a great extent	Some what	Very little	Not at all	Don't know	To a great extent	Some what	Very little	Not at all	Don't know	
2.2.1 Currently, all thoracic centers have timely access to diagnostics so that all testing (e.g. PET scan, CT, percuta-neous biopsies, bronchoscopy and EBUS, cranial imaging etc.) can be completed within defined wait times for cancers..											
Currently, it is the joint responsibility of the region, institution and surgeon to provide appropriate supports and timely access to services (from suspicion to diagnosis to treatment).											
2.2.2 The following resources/collaborating services currently exist or are being used for thoracic surgery patients to provide comprehensive and timely care.<br><br>a) Dedicated geographically											

defined thoracic surgical unit with a consolidated unit of dedicated beds for all elective and emergency cases to ensure an appropriate level of nursing, physiotherapy, and respiratory therapy expertise;<br>b) Step-down beds when necessary;<br>c) 24 hours a day/7 days a week access to the operating room, interventional radiology and critical care;<br>d) access to rapid response laboratory (i.e. biochemistry, cytology, hematology, transfusion and microbiology) services;<br>e) onsite pathology and frozen sections to support operative room;<br>f) timely access to appropriate immunohistochemistry and genomics;<br>g) access to advanced endoscopy (flexible and rigid, EBUS, EUS, stenting) and ambulatory services including interventional endoscopy with the inclusion of ablation and/or mucosal resection.			
2.2.3 Currently, all thoracic centers have well-maintained and adequately resourced open, minimally invasive and advanced endoscopic equipment.			
2.2.4 Currently, capital expenditures are available to provide contemporary equipment and are re-evaluated as there are changes in			

workforce.			
2.2.5 Currently, all thoracic-related pathology reports should be reported in a synoptic format and are completed and communicated within 2 weeks of operation.			
Currently, all thoracic-related pathology reports are completed and communicated within 2 weeks of operation.			

2.3 HUMAN RESOURCES

Standards	Your Institution					Province					Comments
	To a great extent	Some what	Very little	Not at all	Don’t know	To a great extent	Some what	Very little	Not at all	Don’t know	
2.3.1 Currently, thoracic surgery patients are treated by a multi-disciplinary team of advanced health care professionals and human resource supports including, but not limited to respiratory therapists; dietary and nutritional support; home care and social work; allied health professionals such as physician assistants, nurse practitioners and advanced practice nurses at each thoracic center with adequate numbers to support care of patients; allied health support staff including dedicated thoracic nurses and chest physiotherapists available 7 days a week; ready access to on-site palliative care services; thoracic anesthesiologists, pathologists and											

radiologists with an interest in thoracic surgery (preferably with thoracic fellow-ship training and/or mentored by professionals experienced in thoracic surgery); formalized partnerships and access to oncology resources including medical oncologists and radiation oncologists; timely access to other medical specialists including gastroenterologists, infectious disease specialists, cardiologists, neurologists, pulmonary medicine specialists, intensivists, thoracic pathology, and radiologists with a subspecialty interest in diagnostic and interventional procedures of the chest; cancer patient navigators/co-ordinators.			

2.4 TREATMENT AT ONCOLOGY CENTRES AND RELATIONSHIP WITH AFFILIATED CENTRES

Standards	Your Institution					Province					Comments
	To a great extent	Some what	Very little	Not at all	Don't know	To a great extent	Some what	Very little	Not at all	Don't know	
2.4.1 Currently, there exists a strong relationship with a cancer center with access to consultation from medical and radiation oncologists. There is a mechanism in place to provide urgent inpatient consultation and treatment.											

3 QUALITY PROCESSES

3.1 MULTIDISCIPLINARY DISCUSSION AND EVALUATION [OF CASES]

Standards	Your Institution	Province	Comments

	To a great extent	Some what	Very little	Not at all	Don't know	To a great extent	Some what	Very little	Not at all	Don't know	
3.1.1 Currently, all complex lung and esophageal cancers and other thoracic malignancies are discussed in a multidisciplinary format with an attending staff thoracic surgeon, medical and radiation oncologist, pathologist , radiologist and/or nuclear medicine physician, to achieve of optimal outcomes.											

3.2 DATA COLLECTION AND CONTINUOUS QUALITY IMPROVEMENT

Standards	Your Institution					Province					Comments
	To a great extent	Some what	Very little	Not at all	Don't know	To a great extent	Some what	Very little	Not at all	Don't know	
3.2.1 Currently, thoracic surgery centers support quality processes such that social and financial barriers are not a limitation to participation in quality improvement.											
3.2.2 Currently, it is the joint responsibility of the thoracic surgery center and thoracic surgeons to actively monitor patient complications, identify adverse events, and have human resources and processes in place to support quality improvement.											
3.2.3 Currently, there is implementation of routine prospective data collection on process and outcomes such that a data driven approach is used to deliver best practice in care. This includes systematic classification of											

adverse events, regular review of morbidity and mortality rounds, and periodic review of data to allow for self-evaluation and to promote continuous cyclical improvement (through audit and feedback).			
3.2.4 Currently, institutions support adequate collection and measurement of patient experience data.			
3.2.5 Currently, when new technologies and/or processes of care are adapted, there is active tracking of adverse events and outcomes will be completed.			
3.2.6 Currently, institutions have ready access to smoking cessation supports and surgeons actively encourage or refer patients to smoking cessation programs.			
3.2.7 Currently, there exists systematic communication and documentation tools, in alignment with published best practice guidelines, embedded into quality processes to minimize errors in care and enhance quality of care delivered to patients.			

Primary Local, Regional and Provincial Contact Information:

Please provide the names and contact information for representatives in your province with the authority and responsibility to improve surgical care at the local, regional and provincial level.

Local

Regional

Provincial

Are you willing to participate in initiatives to help improve compliance and implementation of the above standards in your province?

1. Yes
2. No

Table S2: Table of Survey Results for Standards with Lowest Implementation for Each Institution and Province

ANALYSIS BY Institute and Province **PERCENTAGES**

Standard	Your Institution					Your Province				
	To a great extent	Somewhat	Very little	Not at all	Don't know	To a great extent	Somewhat	Very little	Not at all	Don't know
**Category 1: Collection, measurement of data related to patient experience, quality, and best practices**										
3.2.3 Currently, there is implementation of routine prospective data collection on process and outcomes such that a data driven approach is used to deliver best practice in care. This includes systematic classification of adverse events, regular review of morbidity and mortality rounds, and periodic review of data to allow for self-evaluation and to promote continuous cyclical improvement (through audit and feedback). a) Your institution	32%	43%	16%	8%	0%	0%	14%	22%	8%	56%
3.2.4 Currently, institutions support adequate collection and measurement of patient experience data. a) Your institution	6%	43%	26%	23%	3%	0%	24%	15%	6%	55%
3.2.7 Currently, there exists systematic communication and documentation tools, in alignment with published best practice guidelines, embedded into quality processes to minimize errors in care and enhance quality of care delivered to patients. a) Your institution	3%	54%	24%	14%	5%	0%	22%	14%	3%	61%
**Category 2: Regional planning and needs assessment for thoracic surgeons**										
2.1.1 a) Recognizing regional needs, currently, a thoracic centre has at least three thoracic surgeons at each designated centre.	86%	0%	0%	11%	3%	58%	0%	0%	39%	3%
b) Currently, recruitment of additional thoracic surgeons is based on certain factors or thresholds such as increase in non-clinical responsibilities (education, research or leadership); workload; referrals; wait times.	51%	35%	0%	11%	3%	28%	36%	0%	11%	25%
c) Currently, recruitment of additional human resource supports is based on certain factors or thresholds such as increase in non-clinical responsibilities (education, research or leadership); workload; referrals; wait times.	19%	36%	19%	11%	14%	6%	24%	6%	9%	56%
**Category 3: Pathology turn-around time and standardized formats**										

2.2.5 a) Currently, all thoracic-related pathology reports should be reported in a synoptic format and are completed and communicated within 2 weeks of operation.	43%	46%	11%	0%	0%	22%	19%	8%	0%	50%
b) Currently, all thoracic-related pathology reports are completed and communicated within 2 weeks of operation.	36%	44%	17%	3%	0%	17%	19%	8%	0%	56%

Questionnaire S2: Consolidated criteria for reporting qualitative studies (COREQ): 32-item checklist[6]

No	Item	Guide questions/description
**Domain 1: Research team and reflexivity**		
Personal Characteristics		
1.	Interviewer/facilitator	Which author/s conducted the interview or focus group?<br>Drs. A. Arnaout and C. Finley conducted the thoracic focus group.
2.	Credentials	What were the researcher's credentials? *E.g. PhD, MD*<br>Dr. A Arnaout: MD, MSc, FRCSC, FACS, MBA<br>Dr. C Finley: MD FRCSC
3.	Occupation	What was their occupation at the time of the study?<br>Dr. Angel Arnaout<br>Expert Lead, Knowledge Mobilization, Canadian Partnership Against Cancer<br>Scientist, Ottawa Hospital Research Institute<br>Surgical Oncologist, Ottawa Hospital<br>Professor, Department of Surgery, University of Ottawa<br><br>Dr. Christian Finley<br>Expert Lead, Clinical Measures, Canadian Partnership Against Cancer<br>Thoracic Surgeon, St. Joseph's Healthcare Hamilton<br>Associate Professor, Department of Surgery, McMaster University
4.	Gender	Was the researcher male or female?<br>Dr. A. Arnaout - Female<br>Dr. C Finley - Male
5.	Experience and training	What experience or training did the researcher have?

No	Item	Guide questions/description
		Dr. Arnaout has research experience in knowledge translation and implementation science, as well as mixed methods and health services research.<br>Dr. Finley has experience in health services research at the policy level.
Relationship with participants		
6.	Relationship established	Was a relationship established prior to study commencement?<br>Drs. Arnaout and Finley were involved in establishing and conducting sessions to develop the pan-Canadian standards for thoracic cancer surgery.
7.	Participant knowledge of the interviewer	What did the participants know about the researcher? e.*g. personal goals, reasons for doing the research*<br>The participants were thoracic surgeons who were invited to participate in the survey and were attending the annual Canadian Association of Thoracic Surgeons meeting. They were aware that the purpose of the focus group was to discuss the categories of surgical standards that were perceived as being the lowest in implementation and to discuss enablers and barriers to their implementation.
8.	Interviewer characteristics	What characteristics were reported about the interviewer/facilitator? e.g. Bias, assumptions, reasons and interests in the research topic<br>A recruitment email was sent to the participants which highlighted that participation was voluntary. Dr. Arnaout was identified as the Principal Investigator. The participants were informed that the Ottawa Health Science Network Research Ethics Board (OHSN-REB) has approved this protocol. Pg. 4 (in accordance with CHERRIES)
**Domain 2: Study design**		The study design was in alignment with Checklist for Reporting Results of Internet E-Surveys (CHERRIES)
Theoretical framework		

No	Item	Guide questions/description
9.	Methodological orientation and Theory	What methodological orientation was stated to underpin the study? *e.g. grounded theory, discourse analysis, ethnography, phenomenology, content analysis*<br>Content analysis was stated to underpin the study. In addition, contextual information was provided to the participants around the Canadian Partnership Against Cancer (CPAC) recently publishing pan-Canadian evidence based surgical standards for thoracic, gynecological, breast and colorectal malignancies. In an effort for quality improvement within clinical practice and across Canada, focus groups were proposed with a representative sample of surgeons from each disease site to further understand the barriers and facilitators to implementation of the CPAC surgical standards.
Participant selection		
10.	Sampling	How were participants selected? *e.g. purposive, convenience, consecutive, snowball*<br>Convenience sampling. P. 4 (in accordance with CHERRIES)
11.	Method of approach	How were participants approached? e*.g. face-to-face, telephone, mail, email*<br>Email
12.	Sample size	How many participants were in the study?<br>15
13.	Non-participation	How many people refused to participate or dropped out? Reasons? None<br>None
Setting		

No	Item	Guide questions/description
14.	Setting of data collection	Where was the data collected? e.*g. home, clinic, workplace*<br>Annual Canadian Association of Thoracic Surgeons (CAGS) meeting on Sept 13-16, 2019
15.	Presence of non-participants	Was anyone else present besides the participants and researchers?<br>Canadian Partnership Against Cancer support staff
16.	Description of sample	What are the important characteristics of the sample? *e.g. demographic data, date*<br>Thoracic surgeons, stratified by Canadian province or territory
Data collection		
17.	Interview guide	Were questions, prompts, guides provided by the authors? Was it pilot tested?<br>Yes, there were questions used to facilitate discussions for the focus group. The questions were reviewed and validated prior to being used for facilitation.
18.	Repeat interviews	Were repeat interviews carried out? If yes, how many?<br>No
19.	Audio/visual recording	Did the research use audio or visual recording to collect the data?<br>Digital recordings from the focus groups were transcribed verbatim, imported into the qualitative data analysis software NVivo (version 1033) and verified by the study team prior to analysis.
20.	Field notes	Were field notes made during and/or after the interview or focus group?<br>During and after the focus group
21.	Duration	What was the duration of the interviews or focus group?<br>The focus group lasted 1.5 hours.

No	Item	Guide questions/description
22.	Data saturation	Was data saturation discussed?<br>Yes
23.	Transcripts returned	Were transcripts returned to participants for comment and/or correction?<br>No
**Domain 3: Analysis and findings**		
Data analysis		
24.	Number of data coders	How many data coders coded the data?<br>4
25.	Description of the coding tree	Did authors provide a description of the coding tree?<br>Yes
26.	Derivation of themes	Were themes identified in advance or derived from the data?<br>Themes were derived from the data.
27.	Software	What software, if applicable, was used to manage the data?<br>NVivo
28.	Participant checking	Did participants provide feedback on the findings?<br>No
Reporting		
29.	Quotations presented	Were participant quotations presented to illustrate the themes / findings? Was each

No	Item	Guide questions/description
		quotation identified? e.*g. participant number*<br>No
30.	Data and findings consistent	Was there consistency between the data presented and the findings?<br>Yes
31.	Clarity of major themes	Were major themes clearly presented in the findings?<br>Yes.
32.	Clarity of minor themes	Is there a description of diverse cases or discussion of minor themes?<br>Yes.

Questionnaire S3: Abridged Focus Group Questions

1. Now that you have seen the survey results of the standards, let’s focus on the certain standards that show a wide gap between evidence and practice. For this standard X, we would like for each of you to tell us about your practice related to this standard. For example, is this standard something you follow in your day to day practice? Why or why not?

Facilitators

2. Now we are going to talk about the factors in your institution that you feel help facilitate your daily practice. What organizational, managerial, or resource factors help you practice in the way you wish to practice?

3. Now we are going to discuss a different type of facilitator – personal facilitators. When you look at your colleagues, what personal factors, that is, their personalities, beliefs, education, or experiences, do you think help them to optimize their use of standardized, timely assessment and effective interventions?

Barriers

4. We will now shift gears and talk about the barriers that hinder our practice, making it different from our desired practice. What aspects of your institution, its policies and procedures, the management, or other health care workers, act as barriers to you or your colleagues to implement the standards?

5. Now we are going to discuss a different type of barrier – personal barriers. When you look at your colleagues, what personal characteristics, that is, personalities, beliefs, education or experiences, etc., hinder their practice?

Opportunities, Strategies and interventions

6. Now I would like you to switch your thinking and remember some different strategies that you have found useful. What strategies have you been exposed to help implement the standard and and what strategies did not?

7. You have all mentioned many barriers and facilitators to your practice related to this standard X. Keeping these in mind, please share your opinion on what an ideal intervention is best geared toward increasing a clinician use of the standard. What would effective interventions would look like? What would be the target audiences and how would it be conducted? Are there any additional strategies that you think may be useful?
